# Rocky Mountain Spotted Fever in Children along the US‒Mexico Border, 2017–2023

**DOI:** 10.3201/eid3011.231760

**Published:** 2024-11

**Authors:** Leslie Chiang, Nanda Ramchandar, Jacquelyn Aramkul, Yaron Fireizen, Mark E. Beatty, Madeleine Monroe, Seema Shah, Jennifer Foley, Nicole G. Coufal

**Affiliations:** University of California San Diego, San Diego, California, USA (L. Chiang, N. Ramchandar, Y. Fireizen, J. Foley, N.G. Coufal); Naval Medical Center San Diego, San Diego (N. Ramchandar); Rady Children’s Hospital of San Diego, San Diego (N. Ramchandar, Y. Fireizen, J. Foley, N.G. Coufal); County of San Diego Health and Human Services Agency, San Diego (J. Aramkul, M.E. Beatty, M. Monroe, S. Shah)

**Keywords:** Rocky Mountain spotted fever, rickettsia, bacteria, vector-borne infections, next-generation sequencing, cell free microbial DNA, children, United States, Mexico

## Abstract

Rocky mountain spotted fever (RMSF) causes significant illness and death in children. Although historically rare in California, USA, RMSF is endemic in areas of northern Mexico that border California. We describe 7 children with RMSF who were hospitalized at a tertiary pediatric referral center in California during 2017–2023. Five children had recent travel to Mexico with presumptive exposure, but 2 children did not report any travel outside of California. In all 7 patients, *Rickettsia rickettsii* DNA was detected by plasma microbial cell-free next-generation sequencing, which may be a useful diagnostic modality for RMSF, especially early in the course of illness, when standard diagnostic tests for RMSF are of limited sensitivity. A high index of suspicion and awareness of local epidemiologic trends remain most critical to recognizing the clinical syndrome of RMSF and initiating appropriate antimicrobial therapy in a timely fashion.

Rocky Mountain spotted fever (RMSF) is a life-threatening infection caused by the tickborne pathogen *Rickettsia rickettsii*. The estimated overall case-fatality rate of RMSF is 7%–10% in the United States (*1*); however, case-fatality rates are considerably higher and approach >50% in several states along the US‒Mexico border (*2*). Diagnosing RMSF requires a high degree of clinical suspicion; the organism is rarely isolated using standard culture methods, and ≈50% of case-patients do not report a history of tick bite (*3*). Moreover, diagnosis can be especially challenging in historically nonendemic regions because many of the signs and symptoms overlap with those of other infectious diseases. Serologic testing, PCR, immunostaining, or specialized culture methods may be used to diagnose RMSF on specimen types including whole blood or biopsy or autopsy tissue and are available through commercial, state health department, or Centers for Disease Control and Prevention (CDC) laboratories (*1*). Indirect immunofluorescence antibody (IFA) assays are a commonly used and widely commercially available diagnostic method; however, antibodies may not develop until the second week of illness (*1*). Prompt empiric administration of effective antimicrobial therapy is critical to averting illness and death. 

Although historically rare in California, RMSF is endemic across areas of northern Mexico; since 2008, RMSF has reached epidemic proportions (*2*,*4*) with especially high case-fatality rates among children (*5*). Most cases of RMSF in southern California are associated with travel to Mexico; a recent update from the Centers for Disease Control and Prevention Health Alert Network reported 5 cases of severe RMSF during July‒December 2023 with recent travel to Tecate, Mexico (*6*). Four of the 5 case-patients were children, and 4 case-patients died. 

We describe 7 children with RMSF diagnoses in San Diego and in whom *R. rickettsii* was detected by plasma microbial cell-free next-generation sequencing (mcf-NGS). This study was approved by the Human Research Protections Program at the University of California, San Diego.

## Methods

We conducted a retrospective chart review of case-patients who had a diagnosis of RMSF during January 1, 2017‒August 30, 2023, and were admitted to Rady Children’s Hospital (San Diego, CA, USA), a tertiary children’s hospital with a broad referral base extending throughout the greater San Diego region. We obtained data from review of electronic medical records. All mcf-NGS testing (Karius Inc., https://kariusdx.com) was sent at the clinical discretion of the treating infectious disease physicians. Cases were confirmed by either serology or PCR testing (Quest Diagnostics, https://www.questdiagnostics.com) and cross-referenced with cases reported to San Diego County Health and Human Services Agency to ensure that no additional pediatric cases occurred during the review period. There were no pediatric cases of RMSF in San Diego for which mcf-NGS was not sent or where mcf-NGS was negative but other testing was positive for RMSF. 

## Results

Seven cases of RMSF were diagnosed in the study period. There were no patients within the study period who had RMSF for whom plasma mcf-NGS was not obtained. The case-patients were 17 months to 14 years of age; mean age was 7.3 years and median 7 years (interquartile range [IQR] 4–10 years). Four case-patients (57%) were male and 3 (43%) were female. Case-patients had a median of 6 days of symptoms (range 5–7 days) before hospital admission. Care was sought for all case-patients during February‒October (Table).

**Table:** Epidemiologic details for 7 cases of Rocky Mountain spotted fever at the US–Mexico border among children treated at Rady Children’s Hospital, San Diego, California, USA

Case	Age/sex	Month of illness	Residence	Travel to/within Baja California, Mexico, in 14 d preceding admission	History of tick bite	History of other animal exposure
1	5 y/M	April	Tecate, Mexico	Primary residence in Tecate, Mexico	Yes	Dogs
2	10 y/F	June	Calexico, CA, USA	To Ensenada, Mexico	No	Goats, pigs
3	7 y/M	February	San Diego, CA, USA	None	No	Dogs
4	14 y/F	October	Mexicali, Mexico	Primary residence in Mexicali, Mexico	Yes	Dogs
5	10 y/F	July	El Centro, USA	To Mexicali, Mexico	No	Dogs
6	17 mo/M	August	El Cajon, USA	To Tecate, Mexico	No	Dogs
7	4 y/M	June	Alpine, CA, USA	None	No	Cats, chickens

Six case-patients were critically ill and required admission to the pediatric intensive care unit (PICU). All case-patients had *R. rickettsii* detected by plasma mcf-NGS; 6 had the diagnosis of RMSF confirmed with serology and 1 by PCR, although 3 initially did not have detectable rickettsial antibodies (Appendix Table). The median time from specimen collection to result return was 3.3 days (IQR 3–3 days) for plasma mcf-NGS and 3.9 days (IQR 2.5–5 days) for serologic testing. Plasma PCR for *R. rickettsii* was sent in 1 case and the result received in 3 days. Antimicrobial therapy was altered because of the mcf-NGS result in 4/7 cases.

### Case 1

**Figure 1:**
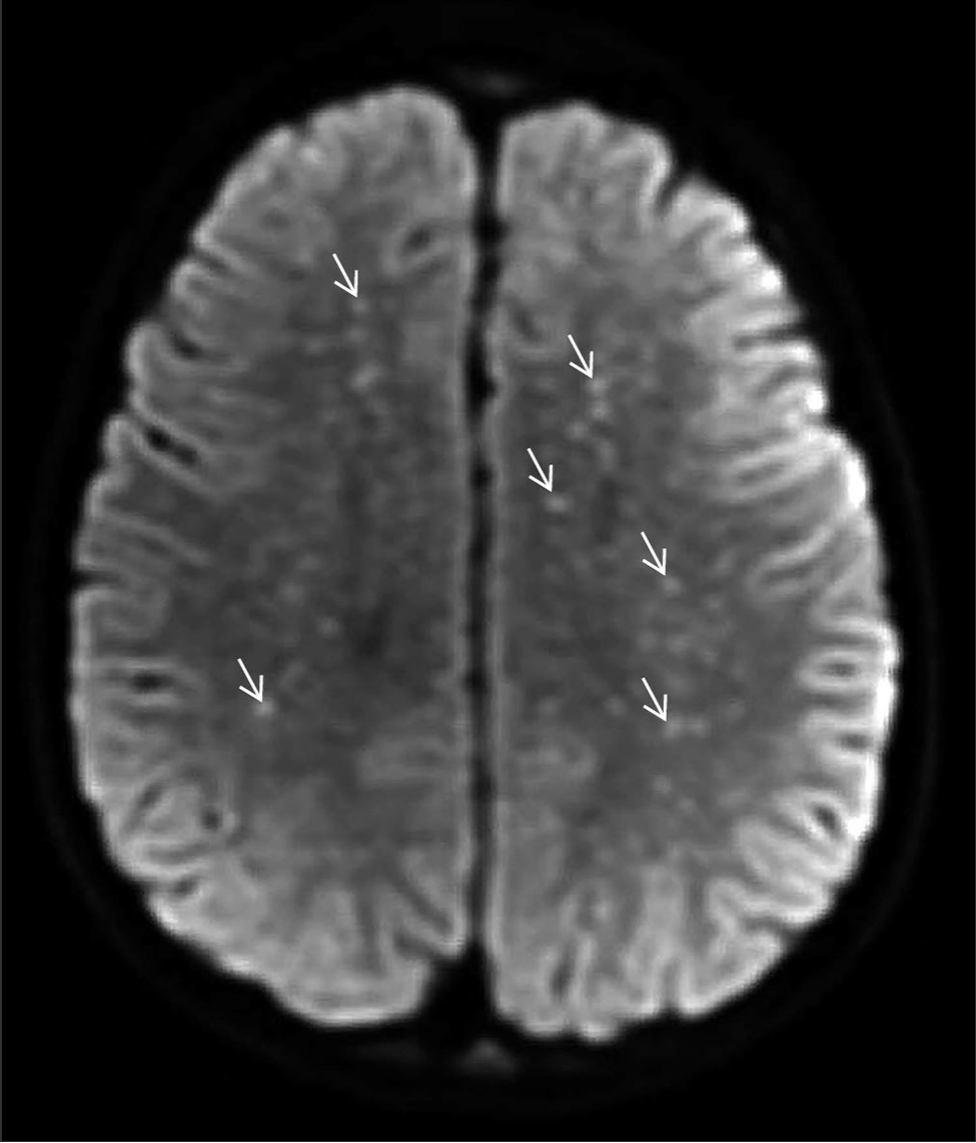
Diffusion-weight magnetic resonance imaging of the brain of a previously healthy 5-year-old boy from Tecate, Mexico, who had Rocky Mountain spotted fever and was treated at Rady Children’s Hospital, San Diego, California, USA. The “starry sky” appearance shows numerous punctate foci of cytotoxic edema involving the supratentorial white matter (arrows), in keeping with acute to subacute injury from small vessel vasculitis.

A previously healthy 5-year-old boy residing in Tecate was brought to the emergency department of Rady Children’s Hospital for 5 days of fever, abdominal pain, vomiting, rash, and altered mental status. The rash began on his face and extremities and subsequently generalized to the entire body. His family recalled a tick bite on his neck ≈2 weeks earlier. Physical examination disclosed a lethargic child with nystagmus, nuchal rigidity, and a diffuse petechial rash that was present on the palms and soles. The patient was admitted to the pediatric intensive care unit. Cerebrospinal fluid (CSF) analysis revealed elevated protein (175 mg/dL; reference range 15–40 mg/dL), glucose level within reference range, and CSF pleocytosis with 53 nucleated cells/μL (reference range 0–10 cells/μL), consisting of 47% neutrophils, 30% mononuclear cells, and 7% lymphocytes. Empiric ceftriaxone, vancomycin, and doxycycline were initiated. Magnetic resonance imaging (MRI) of the brain was notable for innumerable punctate foci of cytotoxic edema involving the supratentorial white matter (Figure 1). Rickettsial antibodies and plasma mcf-NGS were tested on hospital day (HD) 1. On HD3, mcf-NGS result returned with detection of *R. rickettsii*. Initial rickettsial antibodies results were negative, but plasma PCR tested on HD1 returned positive results on HD5. Vancomycin and ceftriaxone were discontinued on HD3, and doxycycline monotherapy was continued to complete a 19-day course. Follow-up serologic testing in the convalescent stage was not performed. The patient survived, with persistent deficits in gross motor function and speech with plans for ongoing rehabilitation.

### Case 2

**Figure 2:**
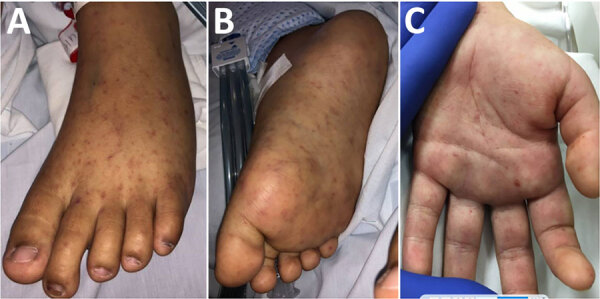
Characteristic petechial rash on the palms and soles of children with Rocky Mountain spotted fever treated at Rady Children’s Hospital, San Diego, California, USA.

A previously healthy 10-year-old girl was brought for care for 7 days of fever, rash, and bilateral conjunctival injection. The rash began on the trunk and spread distally and involved the palms and soles (Figure 2). There was no history of insect bites or tick exposure. The patient lived in Calexico, California, but frequently traveled across the US‒Mexico border, including recent travel to Ensenada in Baja California, Mexico. She was admitted to the pediatric intensive care unit for altered mental status and hypotension, and empiric vancomycin, meropenem, and doxycycline were initiated. Results of CSF analysis, other than an elevated protein of 72 mg/dL, were unremarkable. Results of MRI of the brain were unremarkable. Plasma mcf-NGS and rickettsial serology were tested on HD1; both tests had results on HD3. Mcf-NGS detected *R. rickettsii*; the finding was concordant with the *R. rickettsii* IgM. Vancomycin and meropenem were discontinued; the patient completed an 18-day course of doxycycline. The patient’s altered mental status resolved completely before hospital discharge. At follow-up, she had returned to her baseline state of health.

### Case 3

A previously healthy 7-year-old boy from San Diego was admitted to Rady Children’s Hospital for evaluation of 6 days of fever, maculopapular rash, and joint pain in the ankles and feet. The rash first appeared on his face and spread to involve the trunk, palms, and soles. There was no history of tick exposure, and the family reported no recent travel outside of San Diego. Empiric antimicrobial therapy was not started. After 6 days of hospitalization, during which the fever persisted, mcf-NGS tested was performed, and results returned positive for *R. rickettsii* 2 days later. The rash, which was initially maculopapular in morphology, developed into petechiae during the hospital course. Lumbar puncture and central nervous system imaging was not performed. He was started on doxycycline, after which clinical symptoms resolved. The diagnosis of RMSF was confirmed with positive *R. rickettsii* IgG and IgM from testing sent on HD9 and results received 4 days later. He completed 7 days of doxycycline and returned to his baseline level of health.

### Case 4

A previously healthy 14-year-old girl from a rural area of Mexico near Mexicali was admitted to the hospital with rash, fever, and altered mental status. Six days before care was sought, she had a presumed insect bite on her face, which developed a surrounding petechial rash. The rash subsequently generalized to her entire body, including the palms and soles. She was brought to Mexicali General Hospital, where she was stabilized, then transferred to Rady Children’s Hospital for PICU level of care. Empiric vancomycin and ceftriaxone were initiated at Mexicali General Hospital, and doxycycline was initiated upon admission to Rady Children’s Hospital. CSF analysis was notable for elevated protein 277 mg/dL, glucose level within reference range, and CSF pleocytosis with 83 nucleated cells/μL, 85% neutrophils, 12% lymphocytes, and 3% mononuclear cells. Brain MRI showed numerous punctate hemorrhages, thought to reflect small vessel ischemic changes from microvasculitis. Plasma mcf-NGS and rickettsial antibody testing were sent upon hospital admission. Test result for mcf-NGS were positive for *R. rickettsii* on HD4; initial rickettsial antibody testing was negative, but the patient demonstrated seroconversion with detectable *R. rickettsii* IgG antibody on HD7. Vancomycin and ceftriaxone were discontinued on HD4, and she continued doxycycline for a total 14-day course. Once her clinical condition stabilized, her family requested transfer back to the referring hospital to be closer to their home. She displayed significant gross motor, fine motor, and speech deficits at the time of hospital transfer.

### Case 5

A previously healthy 10-year-old girl from El Centro, California, sought care for 5 days of fever, abdominal pain, emesis, dysuria, and rash on her torso, hands, and feet. The patient was initially seen at an outside hospital and noted to have elevated inflammatory markers, hypotension, and coagulopathy, prompting transfer to Rady Children’s Hospital. There was a history of possible exposure to ticks found on the family dog, but the case-patient had not sustained any known tick bites. The family reported recent travel to Mexicali, Mexico. At admission to the pediatric critical care unit, she was started on doxycycline and ceftriaxone. On HD2, she experienced altered mental status and lethargy. Computed tomography (CT) scan of the head showed no acute abnormality. A lumbar puncture showed elevated opening pressure of 26 mm Hg (reference range 7–15 mm Hg). CSF was notable for 48 nucleated cells/μL with 25% monocytes, 34% neutrophils, and 31% lymphocytes and for elevated protein 46 mg/dL; glucose level was within reference range. Testing obtained on HD1 included mcf-DNA, which detected *R. rickettsii*; the antibodies initially obtained from serum were negative. Serologic testing by IFA was repeated on HD7, revealing IgG and IgM reactive with *R. rickettsii*. The results became available 12 days after initial evaluation and 2 days after hospital discharge. She received 7 days of empiric ceftriaxone for a possible urinary tract infection and 14 days of doxycycline. Upon discharge, the patient was at baseline with no neurologic sequelae.

### Case 6

A previously healthy 17-month-old boy from Tecate, Mexico, was brought for care with 1 week of fever and 3 days of lethargy, as well as 5 days of a diffuse maculopapular rash. At the emergency department, he was noted to have seizure activity with leftward eye and head deviation and a petechial rash on his palms, soles, and mucosal surfaces. He was admitted to the pediatric ICU. The family noted tick exposures around their rural home. Empiric meropenem and doxycycline were initiated. An MRI of the brain noted periventricular and subcortical white matter infarcts secondary to vasculitis. A lumbar puncture obtained at admission had 142 nucleated cells/μL with 61% neutrophils, 14% mononuclear cells, and 25% lymphocytes, elevated protein of 115 mg/dL, and glucose level within reference range. Both RMSF serology and mcf-DNA were sent on HD1, with mcf-DNA noting *Rickettsia* returning on HD3, 2 days before serology results returned. He was treated with 14 days of doxycycline and 5 days of ceftriaxone, followed by 5 days of meropenem, for ongoing fever and presumed culture negative sepsis. He was discharged to home with improved mental status and no focal deficits.

### Case 7

A previously healthy 4-year-old boy from Alpine, California, a small town ≈18 miles north of the California‒Mexico border, sought care for 6 days of fever accompanied by hypotension, abdominal pain, and periorbital and peripheral extremity edema. At the onset of fever, he was seen at an urgent care and prescribed amoxicillin for acute otitis media; 4 days before admission, a rash developed on his thighs and extremities that was initially attributed to drug reaction. The fever and rash persisted, and he came to the Rady Children’s Hospital emergency department on day 6 of illness. He had a history of exposure to farm animals, including dogs and chickens, but no known tick exposure. Empiric ceftriaxone and vancomycin were started, and he was admitted to the pediatric ward. He was escalated to the intensive care unit on HD2 for bilateral pleural effusion and respiratory distress; empiric antimicrobial drugs were broadened to cefepime, doxycycline, and vancomycin upon admission to the PICU. Lumbar puncture and CNS imaging were not performed. Both RMSF serology and mcf-DNA were sent on HD3. Both mcf-DNA noting *Rickettsia* and serology results were returned after patient discharge, 6 days after being sent. He was treated with 10 days of doxycycline and a combined 7 days of cephalosporin therapy for presumed culture negative sepsis. He was discharged to home at his baseline level of health.

## Discussion

We described 7 children with RMSF diagnosis at a tertiary academic center in San Diego, California, USA, in whom plasma mcf-NGS detected *R. rickettsii* over a 7-year period. Four of 7 patients had detectable rickettsial antibody titers on initial visit. All cases were independently verified with serology or PCR during their clinical course.

Although RMSF cases are not commonly diagnosed in southern California, RMSF has reached epidemic proportions in northern Mexico, where the brown dog tick, *Rhipicephalus sanguineus* sensu lato, serves as the principal vector (*7*,*8*). Exposures are occurring in domestic and peridomestic settings within urban centers because *Rh. sanguineus* ticks can complete their entire life cycle with dogs as its primary host (*4*,*9*). During 2020–2021, the overall RMSF case fatality rate was 32% across Baja California; Mexicali had the highest number of cases (*10*). Humans are infected in urban centers because of close contact with stray dogs. Domestic dogs may also become infested with brown dog ticks, and children may be especially at risk because they frequently interact with pet dogs and share peridomestic habitats with pet and stray dogs (*11*,*12*). Stray dogs are rare in southern California because of animal control regulations, and pets are often prophylactically treated to prevent tick infestation, likely decreasing *R. rickettsii* transmission by brown dog ticks in southern California. However, the potential for zoonotic disease spread remains as humans and their pets continue traveling to and from Mexico.

In this report, 5/7 patients reported travel to northern Mexico in the 2 weeks preceding hospital admission. San Diego County and neighboring Imperial County share an international border with Mexico; >100 million crossings were reported in 2022 through the public land ports (*13*). San Diego County exemplifies the fluid nature of RMSF transmission, with travelers frequently crossing and residing on both sides of the border. Of interest, 2 patients in this case series had no reported travel to Mexico. Families may travel with pets or bring dogs across the border, thereby importing *R. rickettsii* into southern California where foci of local infection can be established (*2*,*5*–*10*). Still, because the *Dermacentor occidentalis* tick is the sylvatic species that the California State Public Health Department attributed the source for certain non‒travel associated RMSF cases (*8*,*14*) and because *R. rickettsii* has been detected in tick pools collected by the County of San Diego Vector Control Program as recently as 2023 (J. Aramkul, M.E. Beatty, M. Monroe, unpub. data), advising hikers and others engaged in outdoor activities in the habitat of *D. occidentalis* ticks on tick avoidance and removal is relevant.

The use of paired acute and convalescent specimens obtained 2–6 weeks apart for serologic testing is considered the reference standard for diagnosis of RMSF and achieves >90% sensitivity by day 14 of illness (*1*,*15*). However, patients may not develop detectable antibody titers until 7–10 days after illness onset, and they may die before detectable antibodies have developed (*16*). Given the high degree of virulence of RMSF, recognition of the risk factors and clinical syndrome of RMSF and prompt initiation of appropriate empiric antimicrobial therapy is critical; initial negative antibody testing results should not dissuade clinicians from the diagnosis. Although 5 patients in this case series had no known tick bites, all patients reported exposure to domestic animals, most commonly dogs. All patients in this series experienced fever and rash that involved the palms and soles and had common laboratory abnormalities, including hyponatremia, anemia, thrombocytopenia, transaminitis, and elevated C-reactive protein. 

Physical examination, exposure and travel history, and a high index of suspicion are required for first-line healthcare providers to suspect RMSF. RMSF should be considered in the differential diagnosis for any seriously ill patient with recent travel to northern Mexico, even in the absence of classic risk factors or symptoms. Doxycycline is the treatment of choice in patients of all ages, is safe to administer to patients of any age, and should be initiated as soon as there is a clinical suspicion for RMSF without waiting for laboratory confirmation (*1*,*3*).

Plasma mcf-NGS is a noninvasive method of detecting microbial DNA in patient plasma (*17*). Turnaround times for plasma mcf-NGS approach 24–48 hours, short enough that the technology is an actionable method of achieving a diagnosis. In cases where infection is suspected but standard diagnostic tests fail to identify an infectious etiology, mcf-NGS may provide timely identification of a causative organism. In this single-center case series, the time for plasma mcf-NGS result was approximately half a day faster than serologic testing. Although mcf-NGS appears to be a promising diagnostic method for RMSF, doxycycline treatment should be initiated immediately without waiting for even a rapid diagnostic test result; therapeutic delay increases risk for death (*18*,*19*). Most children in our series were critically ill when they sought care and were started empirically on broad-spectrum antibiotics, including doxycycline. Once RMSF was diagnosed, antimicrobial therapy in 4/7 patients was eventually narrowed to doxycycline alone.

We described 7 children with RMSF who were admitted to our facility over 6.5 years. Our experience aligns with recent epidemiologic trends indicating ongoing high levels of RMSF transmission in multiple border states in northern Mexico. Although our study is limited by its small sample size and retrospective nature, our findings suggest that plasma mcf-NGS may be a useful addition to the diagnostic armamentarium in situations where an infectious cause is suspected but the organism is difficult to isolate through standard methods, or where the clinical manifestation is atypical. This consideration is additionally salient in areas where the etiologic agent may not historically be considered endemic. We highlight the importance of closely monitoring epidemiologic trends in RMSF; early detection and treatment of this disease process are critical in averting serious illness.

Appendix Additional information about Rocky Mountain spotted fever infections in children at the US–Mexico border.
