# Supplementary material for: Rocky Mountain Spotted Fever in Children along the US‒Mexico Border, 2017–2023
Source: Emerg Infect Dis. 2024 Nov;30(11):2288–93. doi: 10.3201/eid3011.231760 (PMC11521158; doi:10.3201/eid3011.231760)
Supplement: Appendix — Additional information about Rocky Mountain spotted fever infections in children at the US–Mexico border. [file 23-1760-Techapp-s1.pdf]

Article DOI: http://doi.org/10.3201/eid3011.231760

EID cannot ensure accessibility for supplementary materials supplied by authors. Readers who have difficulty accessing supplementary content should contact the authors for assistance.

Rocky Mountain Spotted Fever in Children along the US–Mexico Border, 2017–2023

Appendix Table. Clinical details for 7 cases of Rocky Mountain spotted fever at the US–Mexico border*

Case	Clinical symptoms at presentation	Select laboratory abnormalities at admission†	HD of test collection, and initial plasma mcf-NGS result in molecules of *Rickettsia rickettsii* DNA per microliter (MPM)	HD of initial Rickettsial test (serology or PCR) collection and result	Date and result of repeat Rickettsial testing, if performed	Initial antibiotic therapy	Change in clinical management; RMSF treatment and duration
1	Fever, abdominal pain, vomiting, rash, altered mental status	CRP: 25.3 mg/dL<br><br>Sodium:129 mEq/L<br>Platelets: $29 \times 10^3/\mu L$<br>AST: 270 U/L<br>ALT: 79 U/L	HD 1; *Rickettsia rickettsii* DNA detected, but not quantified	HD 1; serology: negative, PCR: positive	Not performed	Ceftriaxone, vancomycin, doxycycline	Doxycycline: 19 days
2	Fever, rash, conjunctival injection	CRP: 20.0 mg/dL<br><br>Sodium:133 mEq/L<br>Platelets: $43 \times 10^3/\mu L$<br>AST: 199 U/L<br>ALT: 141 U/L	HD 1; 20,713 MPM	HD 1; serology: positive	Not performed	Meropenem, vancomycin, doxycycline	Doxycycline: 18 days
3	Fever, rash, joint pain	CRP: 6.7 mg/dL<br><br>Sodium: 128 mEq/L<br>Platelets: $72 \times 10^3/\mu L$<br>AST: 331 U/L<br>ALT: 273 U/L	HD 7; 879 MPM	HD 9; serology: positive	Not performed	None	Doxycycline: 7 days
4	Fever, rash, lethargy	CRP: 24.1 mg/dL<br><br>Sodium: 146 mEq/L<br>Platelets: $87 \times 10^3/\mu L$<br>AST: 219 U/L<br>ALT: 146 U/L	HD 1; 902 MPM	HD 1; serology: negative	HD 7, serology: positive	Ceftriaxone, vancomycin, doxycycline	Doxycycline: 14 days

Case	Clinical symptoms at presentation	Select laboratory abnormalities at admission†	HD of test collection, and initial plasma mcf-NGS result in molecules of *Rickettsia rickettsii* DNA per microliter (MPM)	HD of initial Rickettsial test (serology or PCR) collection and result	Date and result of repeat Rickettsial testing, if performed	Initial antibiotic therapy	Change in clinical management; RMSF treatment and duration
5	Fever, rash, abdominal pain, emesis, dysuria	CRP: 21.5 mg/dL<br>Sodium: 138 mEq/L<br>Platelets: 39 $\times$ $10^3/\mu L$<br>AST: 197 U/L<br>ALT: 74 U/L	HD 1, *Rickettsia rickettsii* DNA detected, but not quantified	HD 1, serology: negative	HD7, serology: positive	Ceftriaxone, doxycycline	Ceftriaxone: 7 days<br>Doxycycline: 14 days
6	Fever, rash, lethargy	CRP: 16.1 mg/dL<br>Sodium: 145 mEq/L<br>Platelets: 36 $\times$ $10^3/\mu L$<br>AST: 123 U/L<br>ALT: 32 U/L	HD 1, 16,164 MPM	HD 1, serology: positive	Not performed	Ceftriaxone, vancomycin, doxycycline	Ceftriaxone: 5 days, followed by 5 days of meropenem for ongoing fever<br>Doxycycline: 14 days
7	Fever, rash, abdominal pain, peripheral and periorbital edema	CRP: 4.3 mg/dL<br>Sodium: 132 mEq/L<br>Platelets: 68 $\times$ $10^3/\mu L$<br>AST: 149 U/L<br>ALT: 76 U/L	HD 3, 6,301 MPM	HD3, serology: positive	Not performed	Ceftriaxone, vancomycin, doxycycline	Ceftriaxone: 2 days, followed by 5 days of cefepime for ongoing fever<br>Doxycycline: 10 days

*ALT, alanine aminotransferase; AST, aspartate aminotransferase; CRP, C-reactive protein; HD, hospital day; mcf-NGS, microbial cell-free next-generation sequencing; RMSF, Rocky Mountain spotted fever.

†Normal ranges: CRP, < 1.0 mg/dL; sodium, 135–145 mEq/L; platelets, 250–450 $\times$ 10^3/uL; AST, 8–33 U/L; ALT, 4–36 U/L.
